# Novel 3-dimensional effective regurgitation orifice area quantification serves as a reliable tool to identify severe mitral valve regurgitation

**DOI:** 10.1038/s41598-024-73264-4

**Published:** 2024-09-27

**Authors:** Tobias Harm, Frederic-Joaquim Schwarz, Monika Zdanyte, Andreas Goldschmied, Livia Baas, Parwez Aidery, Serhii Shcherbyna, Ioannis Toskas, Timea Keller, Isabela Kast, Juergen Schreieck, Tobias Geisler, Meinrad Paul Gawaz, Karin Anne Lydia Mueller

**Affiliations:** Department of Cardiology and Angiology, University Hospital Tübingen, Eberhard Karls University Tübingen, Otfried-Müller-Straße 10, 72076 Tübingen, Germany

**Keywords:** Mitral regurgitation, Effective regurgitation orifice area, 3D EROA, Three-dimensional Echocardiography, Vena Contracta Area, Diagnostic value, Cardiology, Valvular disease

## Abstract

A precise quantification of mitral regurgitation (MR) severity is essential for treatment and outcome of patients with MR. 3D echocardiography facilitates estimation of MR but selection of patients with necessity of invasive treatment remains challenging. We investigate effective regurgitation orifice area (EROA) quantification by 3D compared to 2D echocardiography in patients with MR and highlight the improved discrimination of MR severity. We consecutively enrolled fifty patients with primary or secondary and at least moderate MR undergoing 2D and 3D colour Doppler echocardiography prior to transcatheter edge-to-edge repair (TEER). Improved accuracy of MR grading using 3D vena contracta area (VCA) as an estimate of EROA was compared to 2D proximal isovelocity surface area (PISA) quantification method and a multiparameter reference standard. Quantification of EROA remarkably varies between 2D and 3D echocardiography and the discrimination between moderate and severe MR was significantly (*p* = 0.001) different using 2D PISA or 3D VCA, respectively. 3D VCA correlated significantly (*r* = 0.501, *p* < 0.001) better with the pre-defined MR severity. We detected crucial differences in the correct identification of severe MR between 2D and 3D techniques, thus 2D PISA significantly (*p* < 0.0001) underestimates EROA due to clinical and morphological parameters. The assessment of 3D VCA resulted in improved diagnostic accuracy.

**Supplementary Information:**

The online version contains supplementary material available at 10.1038/s41598-024-73264-4.

## Introduction

Severe MR is a main cause of symptomatic heart failure (HF) necessitating intensive pharmacological and invasive treatment strategies of affected patients^1,2^. Functional MR is a common and critical complication following regional or global left ventricular (LV) dilatation^3^. Likewise, degenerative MR due to abnormalities of the mitral valve leaflets or chordae tendineae is a frequent pathology among adults^4^. Both, severe MR and congestive HF are associated with high morbidity and mortality^3^. However, assessment of MR severity remains challenging and often results in an insufficient therapy of patients with critical MR that comprise adequate pharmacological and invasive treatment strategies including interventional procedures such as percutaneous transcatheter edge-to-edge repair (TEER)^5,6^. Echocardiography is the primary, essential diagnostic tool to assess the severity of MR. Especially functional MR is often underestimated in transthoracic echocardiography and transoesophageal echocardiography can provide many advantages for the correct evaluation of MR^7,8^. Thus, accurate diagnosis of MR and exact grading of MR severity is essential for patient management, treatment, and prognosis^8,9^. According to current guidelines, various non-invasive methods have been implemented to correctly estimate MR severity but a true gold standard in correct diagnostics of severe MR is hitherto lacking in the clinical routine^10^. Additionally, the recommended two-dimensional approach is restricted by indirect estimation of hemodynamic parameters^11^. Thus, addition of 3D-based imaging is a favourable approach to control the major limitations in quantification of MR^12^. Several studies validated the benefit of 3D flow-based vena contracta area (VCA) and proximal isovelocity surface area (PISA) to estimate EROA^7,11,13^. Thus, real-time 3D echocardiography provides a comprehensive visualization of the mitral valve and is the method of choice to precisely grade MR severity^14,15^. Hitherto, the diagnostic value of additional quantification of 3D EROA for grading of severe MR remains unknown. In this study, we aimed to explore the accuracy and diagnostic benefit of three-dimensional echocardiography including 3D VCA in contrast to standard assessment of 2D PISA to estimate EROA. Further, we elucidate the advantages of three-dimensional echocardiography in patients with severe MR in contrast to conventional two-dimensional colour doppler imaging in transthoracic and transoesophageal echocardiography.

## Results

In the present study, we investigate the diagnostic value of three-dimensional echocardiography including the assessment of 3D VCA to estimate EROA in patients with symptomatic, severe MR. The baseline demographic characteristics of the patient cohort are presented in Supplementary Table 1. Twenty-two (*n* = 22, 44%) of the enrolled patients had primary degenerative MR, whereas twenty-eight patients (*n* = 28, 56%) were diagnosed with functional MR. According to the two-dimensional colour Doppler echocardiography pre-defined reference standard, MR of six patients (*n* = 6, 12%) was graded moderate and MR of twenty-three (*n* = 23, 46%) patients was graded moderate to severe. In contrast, twenty-one patients (*n* = 21, 42%) were diagnosed with severe MR. Thirty (*n* = 30, 60%) of the enrolled patients were male, the median age was 81 years [interquartile range (IQR) 77.8–84]. Twenty-two (*n* = 22, 44%) were in atrial fibrillation or atrial flutter during echocardiography with a normal frequency between 60 and 100 bpm. Median LVESD was 41 mm (24.5–47), median LVEDD was 50 mm (42-55.5), and LVEF% was 50 (35–60).

### Comparison of two-dimensional and three-dimensional colour doppler echocardiography

**Fig. 1:**
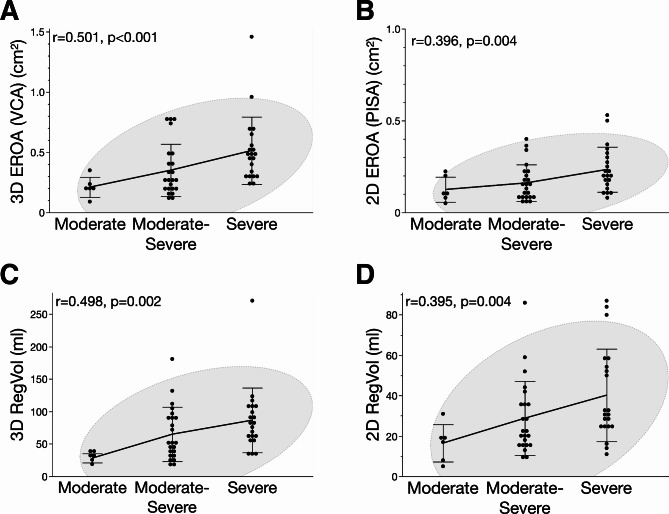
Correlation analysis of quantitative 2D and 3D colour doppler measures and mitral regurgitation severity. Spearman correlation (r) of (**A**) effective regurgitation orifice area (EROA) assessed through 3D vena contracta area (VCA), (**B**) 2D EROA using the proximal isovelocity surface are (PISA) method, (**C**) 2D regurgitation volume (RegVol) and (**D**) 3D RegVol with MR severity as graded by the two-dimensional reference method. All quantitative parameters were significantly (*p* < 0.05) associated with MR severity and 3D EROA showed highest correlation coefficient among all estimators.

MR severity was graded as moderate, moderate to severe or severe, according to the two-dimensional colour Doppler echocardiography reference standard techniques as indicated in the method section. Quantitative colour Doppler parameters of 2D and 3D echocardiography are summarized in Supplementary Table 2. There were significant differences in VCW, 2D EROA by PISA, 3D EROA by VCA and 2D RegVol among moderate, moderate to severe and severe MR grades as classified by the two-dimensional reference standard (*p* < 0.05). It was further noticeable that 3D VCA showed the highest correlation coefficient (*r* = 0.501, *p* < 0.001) among all quantitative colour Doppler parameters assessed in this study (Fig. 1). Further, 3D RegVol (*r* = 0.498, *p* = 0.002), 2D RegVol (*r* = 0.395, *p* = 0.004) and 2D PISA (*r* = 0.396, *p* = 0.004) significantly correlated with the two-dimensional reference method (Fig. 1).

**Fig. 2:**
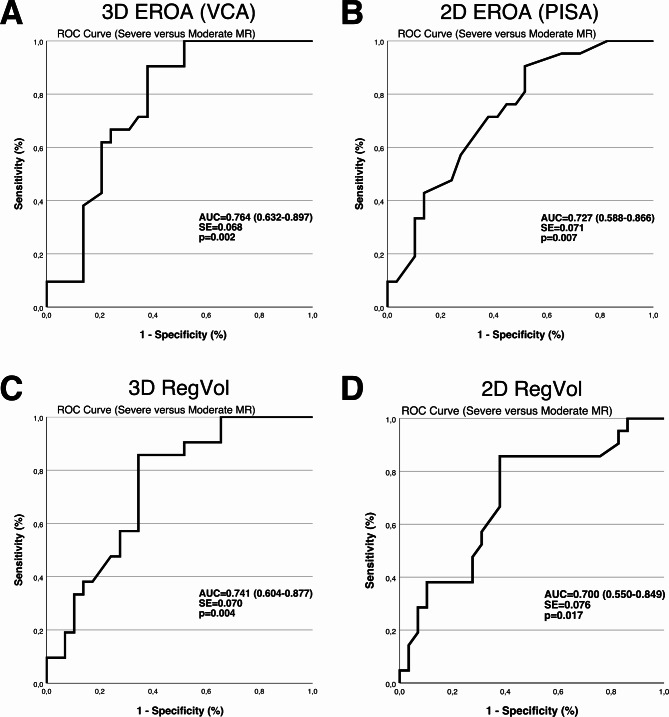
3D EROA improves diagnostic value in patient with severe mitral regurgitation (MR). Receiver operating characteristic (ROC) analysis displaying sensitivity and false positives (1-specificity) to discriminate patients with moderate or severe MR according to the reference method. AUC indicates area under the curve. (**A**) ROC curve shows that 3D effective regurgitation orifice area (EROA) is the most accurate parameter for the detection of severe MR. Further, AUC of (**B**) 2D EROA, (**C**) 3D regurgitation volume (RegVol) and (**D**) 2D RegVol was slightly lower.

To improve the differentiation between severe and moderate MR, ROC analysis was performed, assessing the AUC of distinct quantitative 2D and 3D colour Doppler echocardiography parameters. Here, it was striking that AUC for 3D VCA was 0.764 (95% CI 0.632–0.897, *p* = 0.002) and highest among the tested quantitative parameters (Fig. 2). AUC was 0.741 (95% CI 0.604–0.877, *p* = 0.004) for 3D RegVol, 0.700 (95% CI 0.550–0.849, *p* = 0.017) for 2D RegVol and 0.727 (95% CI 0.588–0.866, *p* = 0.007) for 2D PISA.

### Differences of EROA between 3D and 2D colour doppler echocardiography

**Fig. 3:**
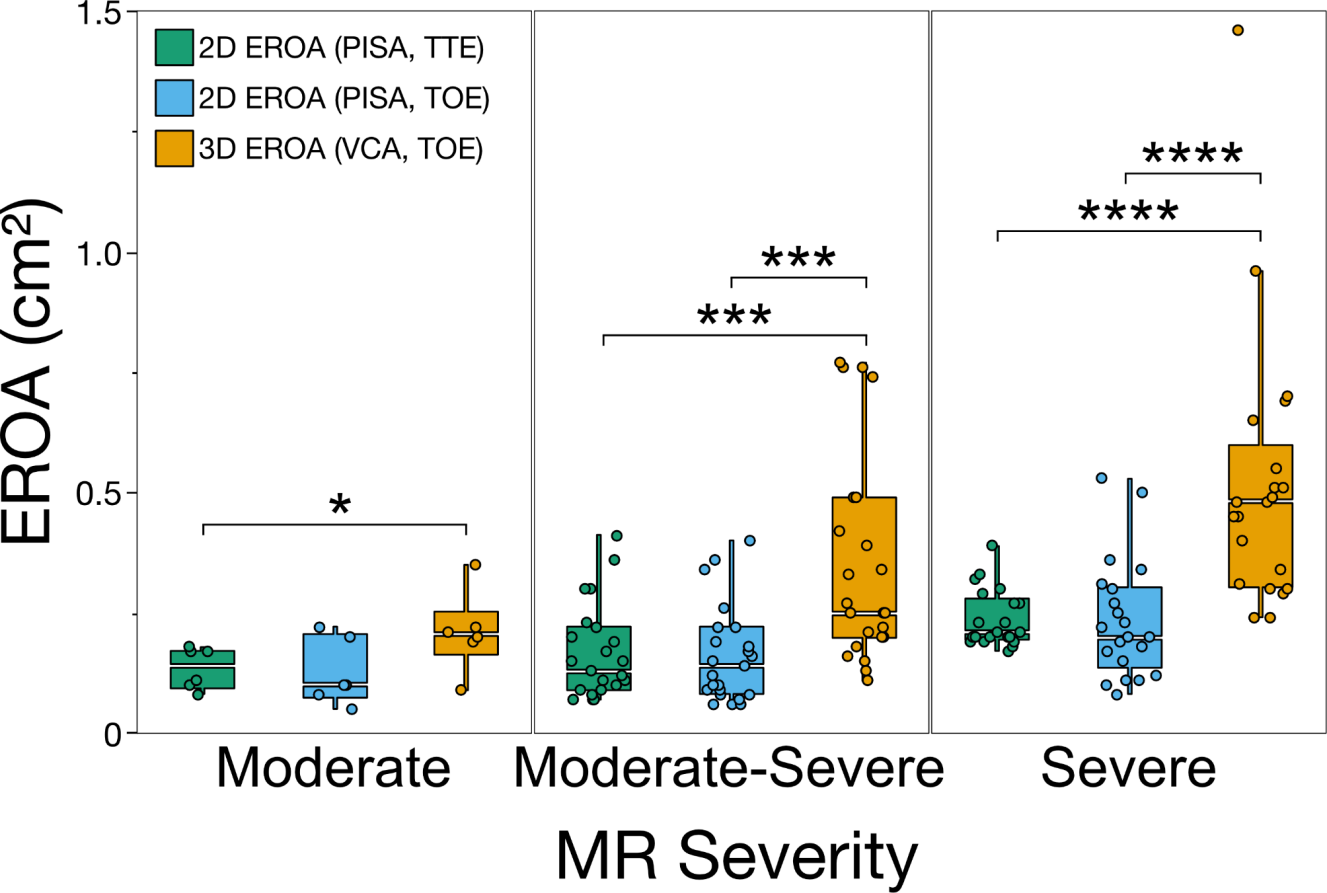
Comparison of 2D and 3D EROA shows significant underestimation by the two-dimensional colour Doppler method. Boxplots displaying the juxtaposition of two- and three-dimensional quantification of effective regurgitation orifice area (EROA, y-axis). X-axis depicts grading of mitral regurgitation (MR) severity according to the reference method. Assessment of EROA by 3D vena contracta area (VCA) was significantly (*p* < 0.05) increased among all grades when compared to the two-dimensional proximal isovelocity surface (PISA) method in both transthoracic (TTE) and transoesophageal echocardiography (TOE). *p < 0.05, **p < 0.01, ***p < 0.001, ****p < 0.0001.

**Fig. 4:**
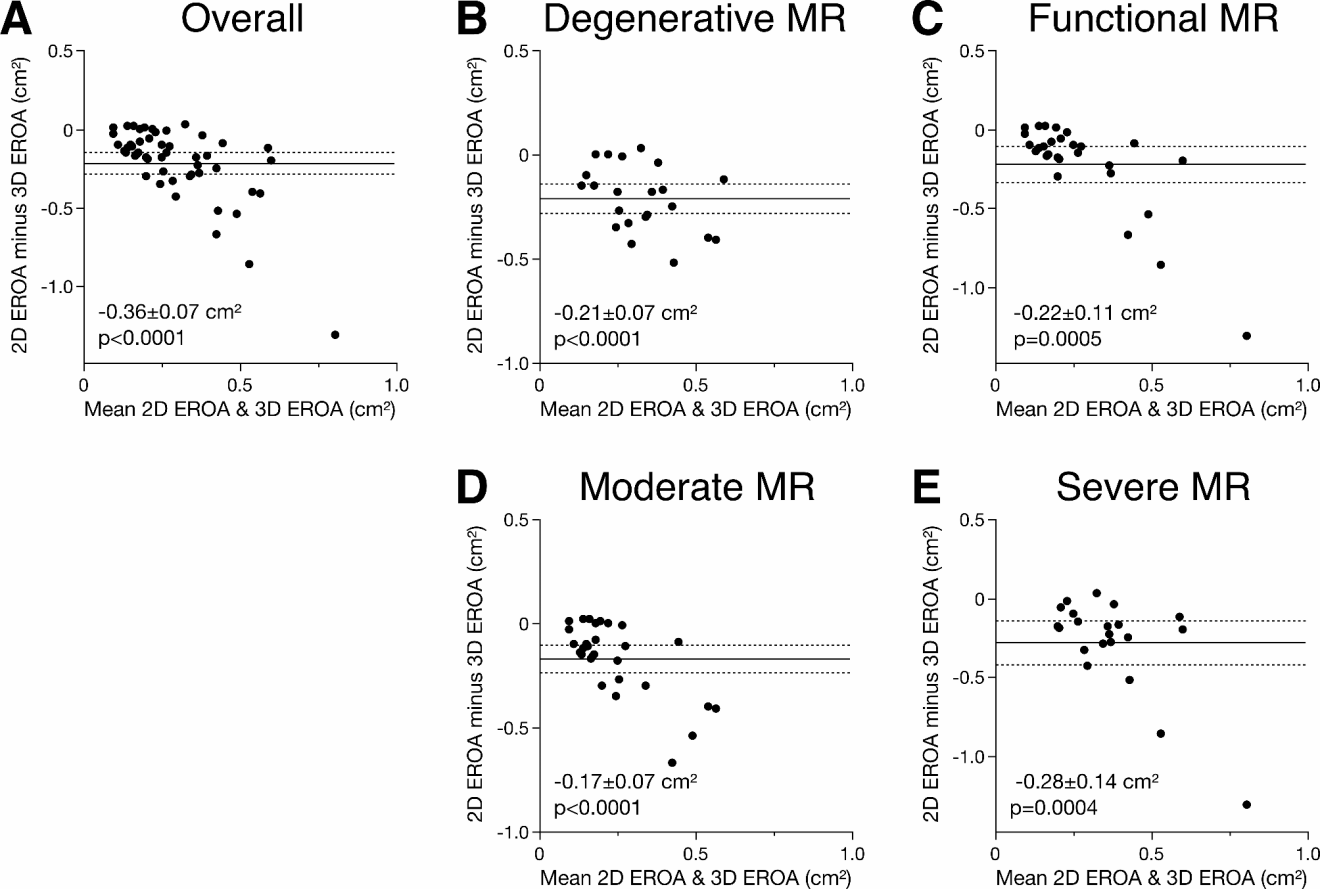
EROA bias of 2D and 3D echocardiography is independent of severity or etiopathogenesis of mitral regurgitation. Bland-Altman plots comparing the 2D PISA quantification method of effective regurgitation orifice area (EROA) and 3D VCA in patients with (**A**) symptomatic mitral regurgitation (MR) and patients with (**B**) primary degenerative and (**C**) functional/secondary MR as well as (**D**) moderate or (**E**) severe MR. EROA was significantly (*p* < 0.05) underestimated by the two-dimensional transoesophageal colour Doppler method when compared to 3D echocardiography.

When comparing two- and three-dimensional quantification of EROA, significant difference among all severity grades were revealed. Quantification of EROA by 3D VCA showed significantly (*p* = 0.045) higher values in patients with moderate MR when compared to 2D PISA method in TTE (Fig. 3). 3D VCA was critically increased in patients with moderate to severe (*p* < 0.001) and severe (*p* < 0.0001) MR when compared to 2D PISA in both TTE and TOE (Fig. 3). This trend was further replicated in Bland-Altman analysis comparing the 2D and 3D quantification of EROA in patient with moderate and severe MR. Comparison of both methods depicted that 3D EROA (VCA) was significantly higher in patients with severe or moderate MR (mean difference − 0.36 ± 0.07 cm^2^, *p* < 0.0001) compared to 2D EROA (PISA) (Fig. 4). The underestimation of EROA by two-dimensional method was consistent among patients with both degenerative and functional MR but did not distinguish between both aetiologies. Mean difference between 2D and 3D EROA was − 0.21 ± 0.07 cm^2^ (*p* < 0.0001) in patients with degenerative MR and − 0.22 ± 0.11 cm^2^ (*p* = 0.0005) in patients with functional MR (Fig. 4). Moreover, the underestimation of EROA by the 2D PISA method was higher in patients with severe MR, as graded by the reference standard, when compared to 3D VCA method (mean difference − 0.28 ± 0.14 cm^2^, *p* = 0.0004) (Fig. 4). Mean difference between both methods was − 0.17 ± 0.07 cm^2^ (*p* < 0.0001) in patients with moderate MR (Fig. 4). These results were consistent in transthoracic echocardiography as 2D PISA method significantly underestimated EROA when compared to 3D VCA assessed through transoesophageal echocardiography (Supplementary Figure S1). The cutoff values proposed by international guidelines at time of study entrance (0.2–0.29 cm^2^, 0.3–0.39 cm^2^, or > 0.4 cm^2^ in degenerative MR and > 0.2 cm^2^ in functional MR) were implemented for the grading of MR severity as moderate, moderate to severe or severe, respectively, using both 2D and 3D EROA. When compared to 2D EROA (PISA) measurements, 56% (*n* = 28) of the patients would have been re-classified to a severe grade, based on the assessment of 3D EROA (VCA). Furthermore, 54.5% (*n* = 12) of the patients with degenerative MR would have been reclassified to severe MR, whereas 53.6% (*n* = 15) of patients with functional MR would have been re-graded as severe. In case of the current cut-off value (≥ 0,4 cm^2^) for severe functional MR, only six patients (21.4%) would have been underdiagnosed by 2D PISA, and thus reclassified to severe MR according to 3D VCA. To test for underlying confounding factors causing the underestimation of EROA by two-dimensional echocardiography, single-predictor bias regression analysis of echocardiographic and hemodynamic measurements (e.g., VCW, RegVol, transmitral gradient, mean and systolic pulmonary artery pressure, wedge pressure, and v-wave) were performed. Here, none of the parameters was associated with underestimation of EROA by 2D echocardiography (Supplementary Table 3). However, to screen for parameters that were associated with underdiagnosis of MR, univariable regression analysis included age, gender, atrial fibrillation, LVEF, left atrial size, aetiology, and severity of MR according to the reference method as well as anterior to posterior (a.p.) and lateromedial (l.m.) anulus diameter of the mitral valve. Here, we found that a.p. (*p* = 0.036) and l.m. (*p* = 0.014) anulus diameter as well as atrial fibrillation (*p* = 0.043) were independent predictive values for the underestimation of 2D EROA (PISA) compared to 3D echocardiography (VCA) (Supplementary Table 4).

Further, variables were entered as covariates in the L_1_-regularized multivariable LASSO analysis and the model showed that atrial fibrillation and l.m. anulus diameter were the only independent predictors of the underestimation of EROA by 2D compared to 3D colour Doppler echocardiography (Supplementary Figure S2).

### Association of 3D EROA with prognostic echocardiographic and clinical parameters

**Fig. 5:**
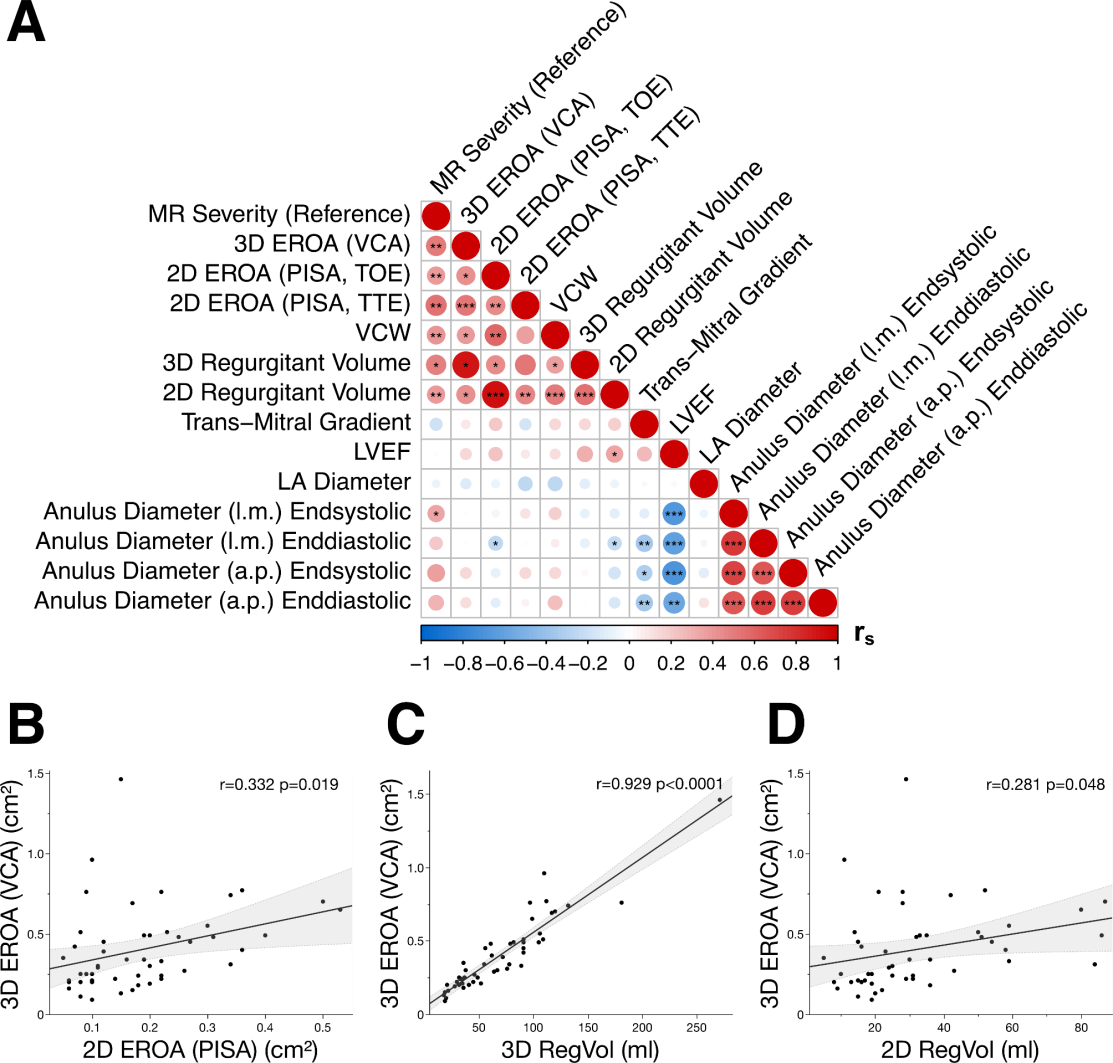
Quantitative colour doppler measurements correlate with clinical parameters in patients with mitral regurgitation. (**A**) Correlation matrix of important quantitative 2D and 3D colour doppler echocardiography estimators in patients with MR. Spearman´s ρ is coloured accordingly and significant (*p* < 0.05) coefficients are labelled (* *p* < 0.05, **p < 0.01, ***p < 0.001). Correlation analysis in patients with mitral regurgitation to evaluate (**B**) the association of 2D PISA and 3D VCA for estimation of effective regurgitation orifice area (EROA) (*r* = 0.332, *p* = 0.019). Correlation analysis of 3D VCA with (**C**) 3D regurgitant volume (RegVol) (*r* = 0.929, *p* < 0.0001) and (**D**) 2D RegVol (*r* = 0.281, *p* = 0.048), accordingly.

As described, quantification of EROA in patients with MR is recommended according to current guidelines and important for exact grading of MR severity. Therefore, we performed comprehensive correlation analysis of important clinical baseline characteristics and quantitative parameters of two- and three-dimensional colour Doppler echocardiography of patients with MR (Fig. 5). We found that 3D VCA significantly correlated with 3D RegVol (*r* = 0.929, *p* < 0.0001), 2D RegVol (*r* = 0281, *p* = 0.048), and 2D PISA (*r* = 0.332, *p* = 0.019), respectively. (Fig. 5). Correlations were homogeneous for both degenerative and functional MR. Interestingly, correlation between 3D and 2D EROA was stronger in patients with degenerative MR (*r* = 0.490, *p* = 0.021), whereas association of 3D VCA and 3D RegVol was highest in patients with functional MR (*r* = 0.932, *p* < 0.0001). It was also noticeable, that important echocardiographic parameters, such as 3D VCA and 2D PISA, were significantly (*p* < 0.05) interrelated (Fig. 5). Additionally, echocardiographic estimates of MR severity were associated with important clinical parameter such as comorbidities, symptoms (i.e. NYHA classification), prognostic risk scores and additional instrumental diagnostics (Supplementary Figure S3).

## Discussion

In our study we could elucidate that: (i) quantification of EROA significantly varies between 2D and 3D echocardiography in patients with moderate to severe MR undergoing TEER, (ii) 2D colour Doppler echocardiography significantly underestimates EROA, (iii) mitral valve anulus diameter and atrial fibrillation independently affect underestimation of EROA, (iv) 3D EROA is associated with distinct 2D and 3D echocardiographic and clinical parameters, iv) quantification of EROA by 3D VCA compared to 2D PISA improves grading and management of patients with severe MR independent of aetiology, and (v) assessment of 3D VCA enhances the diagnostic accuracy of patients with MR and might improve default colour Doppler echocardiography in clinical routine.

Mitral valve regurgitation is a common cause of symptomatic heart-failure leading to decompensation, hospitalization, and unfavourable prognosis if left untreated. The severity of MR accounts for clinical relevance of symptoms, complications and outcome and thus represents an important target for an interventional and pharmacological treatment approach^6,9^. Therefore, precise grading and critical assessment of patients with severe MR is a cornerstone for further sufficient patient management. Hitherto, a combination of two-dimensional colour Doppler echocardiography is the reference method to determine the degree of MR, but exact grading of MR severity remains challenging in clinical practice^1,15^. Transthoracic and transoesophageal echocardiography are the main essential diagnostic tools to evaluate not only morphological features of the mitral valve but also the extent of MR. Conventional reference standard evaluation of MR comprises 2D techniques with 2D colour doppler, width of vena contracta, 2D regurgitation volume, and finally estimation of 2D EROA^10,16^. There is preliminary data suggesting that the addition of quantitative three-dimensional echocardiographic parameters improves the diagnostic value of echocardiography in patients with severe MR^13,14^. However, the importance of 3D EROA in correct grading of clinically relevant MR and efficient selection of patients with severe MR for interventional therapy remains unclear. Therefore, we hypothesized that the assessment of EROA by 3D VCA improves the diagnostic precision of echocardiography compared to the two-dimensional standard method. Conventional 2D echocardiography is limited by indirect assessment of mitral valve geometry, inaccurate hemodynamics and quantification of MR jet parameters^17^. The 2D PISA method provides an indirect measurement of EROA, thus assuming a hemispherical shape resulting in underestimation bias of severe MR and inadequate quantification in patients with discordant anatomy^18,19^. Subsequently, recent studies showed that three-dimensional echocardiography including 3D VCA improves the diagnostic value and might become a new approach for MR quantification by direct measurement of the EROA^13,20^. The 3D echocardiography method is strengthened by the direct measurement of the EROA, and geometric assumptions are discarded, thus potentially resulting in a more accurate assessment of the EROA.

In our study, we highlight that the assessment of 3D VCA is a powerful method for adequate quantification of severe MR and thus is highly associated with the 2D reference standard colour Doppler method. A precise differentiation between severe and non-severe MR is indispensable for patient management, treatment, and prognosis. In particular, grading of MR severity critically influences surgical or catheter-based interventions including TEER. This is especially relevant in patients with severe but asymptomatic MR as implicated in the current guidelines^1,21^ to prevent these patients from advancing heart failure due to delayed pharmacological and/or interventional treatment.

Here, we found that 3D VCA had the highest sensitivity and specificity to identify severe MR among all aetiologies of the mitral valve strikingly shown in our ROC analyses. Therefore, the additional assessment of EROA by 3D VCA along with conventional 2D echocardiographic parameters might improve the correct identification of patients with severe MR in the clinical routine. The 3D VCA method correlated well with both the reference standard method and 2D EROA. Furthermore, 3D VCA was positively associated with 2D and 3D regurgitation volume. Due to the direct measurement of the EROA, three-dimensional echocardiography might support the reference method in patients with eccentric or atypical MR jets^13,22^. By applying individual perpendicular cropping, 3D VCA method might be more accurate in settings with difficult anatomy of MR. However, the improvement of sensitivity and specificity was incremental, and a true gold standard is precluded from direct comparison.

The 2D method often underestimates or overestimates the EROA in case of opposed geometric assumptions and mostly functional MR (Supplementary Figure S6)^11^. Our results showed that in contrast to 3D VCA, the two-dimensional method significantly underestimates the orifice area in both, functional and degenerative MR. Single-predictor and multivariable regression analysis highlighted atrial fibrillation and end-diastolic mitral valve anulus diameter as independent predictors of underestimation of EROA by the 2D colour Doppler method. Atrial fibrillation is a common consequence and highly frequent in patients with severe MR and of prognostic relevance after TEER. Mitral valve anulus diameter is associated with disease progression and is an important parameter which should be integrated in the decision making prior to TEER^23,24^. Thus, in patients with severe MR and atrial fibrillation or mitral valve anulus dilatation, inclusion of 3D VCA to the conventional colour Doppler echocardiography might improve the diagnostic value and therapeutic management and should be implemented as routine diagnostic tool. In this study, the 3D method classified more patients with severe MR independent of the controversial cut-off value in functional MR, which was also of clinical relevance, compared to the 2D EROA. Thus, this might grant access to treatment strategies including TEER for patients who were primarily restrained from adequate therapeutical regimens. Previous studies suggested a more offensive grading of MR, thereby lowering the cutoff values for 2D EROA to improve clinical outcome in patients with severe MR^13^. However, due to the missing true gold standard in quantification of severe MR and preliminary prospective data, upgrading of MR degree without reliable measure tools might lead to an overtreatment in some patients, where the watch and wait strategy would be more appropriate for optimal patient management and improvement of their prognosis.

The validity and significance of our presented analysis is constricted by the rather preliminary, observational study design applied for hypothesis generation as well as by the missing “true gold standard” to warrant qualitative analyses.

Additionally, the cohort size is limited and the number of patients with varying MR gradings and etiologies is modest. This could affect the generalizability of the study, indicating the need for a larger follow-up study to confirm the significance of the results. At present, the novel results of our study are strengthened by the comparison of 3D EROA (VCA) with reliable and prognostic 2D echocardiographic parameters showing a high degree of congruence. However, systolic jet abnormalities and a standardized approach for irregularities of the mitral valve apparatus such as cleft, prolapse or flail leaflet are hitherto missing. Therefore, integrating the effective EROA throughout the cardiac cycle is applied to consider EROA variation and current echocardiography studies recommend the assessment of EROA quantification in case of severe MR^8,10^. The accuracy of 3D VCA assessment is affected by three-dimensional cropping planes. In some patients with eccentric MR or abnormal jet geometry, the measurement of optimal perpendicular plane might lead to under- or overestimation of the effective orifice. However, in regression analysis jet morphology was not associated with underestimation of EROA between 2D and 3D echocardiography. Additionally, volume status and hemodynamic stability can fluctuate before TEER and might affect load-dependent measurements of MR. Independent imaging modalities like cardiac MRI could thus improve the sensitivity of MR severity grading by correlating anatomical features with hemodynamic data. Moreover, inaccuracies of MR grading by 3D VCA might arise from arrhythmias such as atrial fibrillation, respiration, or movement artifacts. Therefore, standardized examination conditions were applied to prevent potential confounding, and atrial fibrillation and mitral valve anatomy were shown to independently predict underestimation of MR severity by conventional 2D echocardiography. However, observed trends of improved diagnostical value by ROC analysis and correlation analysis were incremental and further large-scale studies are needed to confirm the results of beneficial assessment of 3D EROA.

## Methods

### Study population

Fifty (*n* = 50) patients with symptomatic degenerative and functional MR were enrolled in this prospective, consecutive study (Supplementary Table 1). All patients underwent colour Doppler two- and three-dimensional echocardiography with transthoracic and transoesophageal approaches prior to TEER. All patients were treated for symptomatic HF according to international guidelines^1,25^. Exclusion criteria of this study comprised of significant mitral stenosis (area < 2.0 cm^2^), mitral prosthesis or annuloplasty, infective endocarditis, and poor image quality of two- and three-dimensional echocardiography. Two- and three-dimensional colour Doppler echocardiographic examinations were performed by using a Philips EPIQ 7 (Philips Medical Systems, Hamburg, Germany) ultrasound system. All echocardiographic examinations and data acquisition were performed by experienced echocardiographers trained in 2D and 3D echocardiography. The study was approved by the Ethics Committee at the Medical Faculty of the Eberhard Karls University and at the University Hospital of Tübingen (842/2020BO2) and all patients gave written informed consent. The experiments were performed in accordance with the highest ethical standards as laid down in the Declaration of Helsinki.

### Two-dimensional colour doppler echocardiography

All patients underwent 2D echocardiography in left lateral position using a Philips X5-1 (Philips Medical Systems, Hamburg, Germany) (1–5 MHz) array transducer for transthoracic approaches, while transoesophageal echocardiography (TOE) prior to TEER was performed with a Philips X8-2 transducer (Philips Medical Systems, Hamburg, Germany) (1–5 MHz). Nyquist limits ranged from n 30 to 50 cm/ and a colour gain was used to adjust colour speckle artifacts. A breath holding manoeuvre was performed to standardize acquisition of colour Doppler MR jet. LV end-systolic and end-diastolic diameters (LVESD and LVEDD) were quantified by the 2D method from parasternal long-axis view. LV ejection fraction was estimated using the Simpson biplane method. Two-dimensional quantification of MR by TOE included VCW, 2D EROA and 2D RegVol. VCW was measured at the narrowest point of the MR colour Doppler jet in an optimized and magnified view (Supplementary Figure S4). For a more accurate calculation VCW was further quantified in the 3-chamber view in case of asymmetric EROA. The severity of MR using VCW was scored as moderate (0.3 to 0.69 cm) or severe (> 0.7 cm). 2D EROA was obtained using the proximal isovelocity surface area (PISA) method from both transoesophageal and transthoracic echocardiography as indicated^26^. RegVol was estimated as EROA multiplied by the velocity time integral (MR-VTI) of the regurgitation jet.

### Three-dimensional colour doppler echocardiography

3D transoesophageal echocardiography data were obtained from up to 10 consecutive ECG-gated cardiac cycles. In patients with atrial fibrillation, median jet values were calculated from repetitive imaging to mitigate the influence of variable beat length. To maximize the frame rate, the narrowest sector possible was adjusted with Nyquist velocities ranging from 30 to 50 cm/s. The dataset was further analysed offline according to standardized protocols using Philips Qlab 3DQ software. To estimate EROA, direct measurements of VCA were obtained from an en face view using 3D transoesophageal echocardiography, as previously described^26,27^. Here, to obtain 3D EROA two orthogonal image planes and the third perpendicular plane were cropped and calibrated along the jet direction to visualize the cross-section area at the level of the vena contracta (Supplementary Figure S5). 3D EROA was measured by direct planimetry of the colour Doppler flow^26,27^. Severity of degenerative MR was graded as mild (< 0.2cm^2^), moderate (0.2–0.29 cm^2^), moderate-severe (0.3–0.39 cm^2^) or severe (> 0.4 cm^2^) as specified by 3D EROA. When using 3D EROA, functional MR was graded as severe with adapted cut-off values > 0.2 cm^2^ and > 0.4 cm^2^ respectively.

During the implementation of our study, based on two prospective RCTs^28,29^, updated guidelines favoured an increased cut-off value but the definition of severe MR by EROA > 0.4 cm^2^ remains controversial^1^. Therefore, we combined both values for further analysis and applied a lower cut-off to 3D EROA to test for enhanced sensitivity.

RegVol calculation with 3D EROA was carried out similarly to 2D echocardiography by multiplying EROA with MR-VTI.

### Two-dimensional reference method

According to international guidelines, the integrative reference standard for MR grading in this study implemented two-dimensional qualitative, semi-quantitative and quantitative measurements of transoesophageal colour Doppler echocardiography^15,16^. In agreement with current clinical practice, MR grading consisted of analysis of mitral valve morphology and MR jet, VCW, regurgitant fraction of MR derived from indirect estimation of EROA using VCW, jet area to left atrial area ratio and at least one additional parameter (i.e. systolic flow reversal in pulmonary veins, density of MR jet in continuous wave Doppler; left atrial or ventricular enlargement)^13^.

MR was graded moderate to severe if additional parameters matched severe MR but VCV was < 0.7 cm. The two-dimensional reference grading and assessment of 3D EROA were done independently, and the results were blinded, respectively.

### Statistical analysis

Patients baseline characteristics and two- and three-dimensional colour Doppler echocardiography data were analysed using JMP^®^ Version 16.2 (SAS Institute, Cary, North Carolina, USA). Normally distributed data were analysed using student´s t-test, non-normally distributed data were compared using the Mann–Whitney U test and analysis of variance (ANOVA) was used for comparisons of multiple normally distributed data. Mean values are presented as mean ± standard deviation (SD) or as median and interquartile range (IQR) where applicable. Categorical parameters were compared using Chi-Square tests. Correlation data is based on Pearson´s product-moment correlation coefficient and Spearman’s rank correlation coefficient as indicated. Receiver-operator characteristic (ROC) curve for improved prediction of severe MR was calculated for 2D and 3D echocardiography parameters by plotting the dependency of specificity on sensitivity. The ROC curve was computed from a log-linear model and quantified by the area under the curve (AUC). The referring confidence intervals were computed with percentile bootstrap analysis. Agreement between 2D and 3D methods was tested by using Bland-Altman analysis and mean ± 2 SD was plotted. For bias regression, linear regression models assessed the influence of important echocardiographic and hemodynamic characteristics on the difference between estimation of EROA by 2D PISA method compared to 3D VCA. The underestimation of EROA by 2D compared to 3D colour Doppler echocardiography method was calculated as ratio of 2D EROA to 3D EROA and linear regression models were carried out to identify parameters associated with underestimation of EROA by 2D echocardiography. For multivariable analysis all parameters were included, and a penalized least absolute shrinkage and selection operator (LASSO) algorithm was then implemented for variable selection and reduction of data overfitting as described previously^30,31^.

Graphic output was performed with different software packages including R^®^ (R foundation for Statistical Computing, Vienna, Austria) and JMP^®^.

## Supplementary Information

Supplementary Material 1.

## Data Availability

The data that support the findings of this study are available on reasonable request from the corresponding author.
