# Supplementary material for: Novel 3-dimensional effective regurgitation orifice area quantification serves as a reliable tool to identify severe mitral valve regurgitation
Source: Sci Rep. 2024 Sep 27;14:22067. doi: 10.1038/s41598-024-73264-4 (PMC11437129; doi:10.1038/s41598-024-73264-4)
Supplement: Supplementary file 1 — Supplementary Material 1. [file 41598_2024_73264_MOESM1_ESM.docx]

**Novel 3-dimensional effective regurgitation orifice area quantification serves as a reliable tool to identify severe mitral valve regurgitation**

**Short title:** 3D EROA and Severe Mitral Regurgitation

Tobias Harm, Frederic-Joaquim Schwarz, Monika Zdanyte, Andreas Goldschmied, Livia Baas, Parwez Aidery, Serhii Shcherbyna, Ioannis Toskas, Timea Keller, Isabela Kast, Juergen Schreieck, Tobias Geisler, Meinrad Paul Gawaz, Karin Anne Lydia Mueller*

Department of Cardiology and Angiology, University Hospital Tübingen, Eberhard Karls University Tübingen, Otfried-Müller-Straße 10, 72076 Tübingen, Germany

***Correspondence to:**

Karin Anne-Lydia Müller

Department of Cardiology and Angiology

University Hospital Tübingen

Eberhard Karls University Tübingen

Otfried-Müller-Str. 10, 72076 Tübingen, Germany

Tel.: +49 (0) 7071 29 83688

Fax: +49 (0) 7071 29 5749

E-Mail: k.mueller@med.uni-tuebingen.de

**Supplementary Tables and Figures**

**Supplementary Table 1:** **Baseline characteristics of patient population.** Values are presented as median with interquartile range (IQR)

|  | **All Patients (n=50)** |
| --- | --- |
| Female, n (%) | 20 (40) |
| Age, years, median (IQR) | 81 (77.8-84) |
| **Cardiovascular risk factors** |  |
| Arterial hypertension, n (%) | 46 (92) |
| Diabetes mellitus, n (%) | 11 (26) |
| Coronary artery disease, n (%) | 34 (68) |
| Previous myocardial infarction, n (%) | 16 (32) |
| Previous CABG, n (%) | 3 (6) |
| Atrial Fibrillation, n (%) | 22 (44) |
| EuroSCORE II, median (IQR) | 5.4 (2.7-9) |
| STS-Score, median (IQR) | 5.6 (3.9-9.5) |
| **Medication on admission** |  |
| Statins, n (%) | 37 (74) |
| Acetylsalicylic acid, n (%) | 12 (24) |
| Clopidogrel, n (%) | 20 (40) |
| Ticagrelor, n (%) | 3 (6) |
| Prasugrel, n (%) | 0 (0) |
| Oral anticoagulants, n (%) | 36 (72) |
| ACE inhibitors, n (%) | 18 (36) |
| Angiotensin II receptor antagonists, n (%) | 14 (28) |
| Angiotensin receptor-neprilysin inhibitor, n (%) | 6 (12) |
| Aldosterone antagonists, n (%) | 2 (4) |
| Ca^2+^ channel antagonists, n (%) | 12 (24) |
| β-blockers, n (%) | 25 (24) |
| Diuretics, n (%) | 21 (42) |
| **Laboratory parameters and biomarkers** |  |
| Troponin I (mg/dL), median (IQR) | 0.1 (0.1-46.5) |
| CK (mg/dL), median (IQR) | 54 (38.8-87.8) |
| Renal function (GFR), median (IQR) | 55.9 (35.5-79.5) |
| C-reactive protein (mg/dL), median (IQR) | 0.3 (0.1-0.9) |
| Hemoglobin (g/dl), median (IQR) | 11.5 (10-13.3) |
| **Mitral valve regurgitation** |  |
| Degenerative, n (%) | 22 (44) |
| Functional, n (%) | 28 (56) |
| Moderate, n (%) | 6 (12) |
| Moderate-severe, n (%) | 23 (46) |
| Severe, n (%) | 21 (42) |
| **Quantitative echocardiographic parameters** |  |
| Vena contract width (cm), median (IQR) | 5 (4.8-6) |
| Enddiastolic a.p. anulus diameter (mm), median (IQR) | 51 (45-58.2) |
| Enddiastolic l.m. anulus diameter (mm), median (IQR) | 54.2 (46.9-60.1) |
| Systolic flow reversal in pulmonary veins, n (%) | 50 (100) |
| LA diameter (long axis view), median (IQR) | 49.5 (44.8-56.8) |
| LVESD (cm), median (IQR) | 41 (24.5-47) |
| LVEDD (cm), median (IQR) | 50 (42-55.5) |
| Left ventricular ejection fraction (%), median (IQR) | 50 (35-60) |
| PAPsys (mmHg), median (IQR) | 37 (30.5-51.5) |
| **NYHA class** |  |
| I, n (%) | 0 (0) |
| II, n (%) | 2 (4) |
| III, n (%) | 31 (62) |
| IV, n (%) | 17 (34) |

CABG; coronary artery bypass graft; ACE, angiotensin converting enzyme; CK, creatine kinase; LA, left atrium; LVESD, left ventricular endsystolic diameter; LVEDD, left ventricular enddiastolic diameter; a.p., anteroposterior; l.m., lateromedial; PAPsys, systolic pulmonary artery pressure; NYHA, New York Hear Association

**Supplementary Table 2: Comparison of quantitative two- and three-dimensional echocardiography in patients with MR.** The severity of MR was graded according to the reference standard. Significant values (p<0.05, ANOVA) are highlighted

|  | **Moderate MR** | **Moderate-**  **Severe MR** | **Severe MR** | **P Value** |
| --- | --- | --- | --- | --- |
|  | **(n=6, 12%)** | **(n=23; 46%)** | **(n=21; 42%)** |  |
| VCW (cm), mean (±SD) | 5.8 (±1.33) | 6.19 (±2.27) | 7.40 (±2.50) | **0.010** |
| 2D RegVol (ml), mean (±SD) | 16.50 (±9.24) | 28.82 (±18.31) | 40.24 (±22.89) | **0.025** |
| 3D RegVol (ml), mean (±SD) | 28.17 (±7.02) | 65.0 (±41.93) | 119.33 (±155.27) | 0.102 |
| 2D PISA (cm^2^), mean (±SD) | 0.13 (±0.07) | 0.16 (±0.10) | 0.23 (±0.12) | **0.032** |
| 3D VCA (cm^2^), mean (±SD) | 0.21 (±0.03) | 0.35 (±0.21) | 0.51 (±11.7) | **0.012** |

MR, mitral valve regurgitation; PISA, proximal isovelocity surface area; RegVol; regurgitant volume; SD, standard deviation; VCA, vena contract area; VCW, vena contract width.

**Supplementary Table 3: Bias regression for the assessed difference of EROA estimation by 2D PISA method compared to 3D VCA.** Linear regression models were used to identify parameters that were significantly associated (p<0.05) with the discrepancy between 2D and 3D echocardiography.

| **Variables** | **ß Coefficient** | **Std Error** | **R^2^** | **P Value** |
| --- | --- | --- | --- | --- |
| Vena Contracta Width | -0.039 | 0.022 | 0.069 | 0.093 |
| Regurgitant Volume | -0.002 | 0.001 | 0.029 | 0.237 |
| Transmitral Gradient | -0.009 | 0.046 | 0.001 | 0.840 |
| Pulmonary Artery Systolic Pressure | 0.004 | 0.002 | 0.077 | 0.054 |
| Pulmonary Artery Mean Pressure | 0.003 | 0.004 | 0.026 | 0.363 |
| Pulmonary Capillary Wedge Pressure | 0.004 | 0.005 | 0.021 | 0.411 |
| v-Wave | 0.001 | 0.003 | 0.001 | 0.817 |

**Supplementary Table 4: Predictors of underestimation of EROA by 2D compared to 3D colour Doppler echocardiography.** Linear regression models to identify parameters significantly (p<0.05) associated with underestimation of EROA by 2D echocardiography

|  | **Univariable Regression Analysis** | | |
| --- | --- | --- | --- |
| **Variables** | **ß Coefficient** | **Std. Error** | **P Value** |
| Age | -0.08 | 0.01 | 0.582 |
| Gender | -0.20 | 0.09 | 0.160 |
| **Atrial Fibrillation** | 0.14 | 0.07 | **0.043** |
| LVEF | 0.02 | 0.01 | 0.888 |
| LA Size | -0.13 | 0.01 | 0.378 |
| MR Aetiology | 0.01 | 0.09 | 0.927 |
| MR Reference Grade | 0.16 | 0.14 | 0.252 |
| **Anulus Diameter (a.p.)** | -0.22 | 0.11 | **0.036** |
| **Anulus Diameter (l.m.)** | -0.28 | 0.11 | **0.014** |
| Jet Morphology | 0.03 | 0.09 | 0.735 |

LVEF, left ventricular ejection fraction; LVESD, left ventricular end-systolic diameter; LVEDD, left ventricular end-diastolic diameter; LA left atrium; MR, mitral regurgitation; a.p., anteroposterior; l.m., lateromedial

**Supplementary Figure S1. EROA bias is consistent in transthoracic echocardiography (TTE).** Bland-Altman plots comparing the 2D PISA quantification method of effective regurgitation orifice area (EROA) in TTE and 3D VCA from TOE in patients with **(A)** symptomatic mitral regurgitation (MR) and patients with **(B)** primary degenerative and **(C)** functional/secondary MR as well as **(D)** moderate or **(E)** severe MR. EROA was significantly (p<0.05) underestimated by 2D PISA.

**Supplementary Figure S2. Determination of patient characteristics associated with underestimation of EROA by 2D Echocardiography.** A least absolute shrinkage and selection operator regression including important variables from ***supplementary Table 4*** was applied to estimate underdiagnosis of EROA by 2D PISA method compared to 3D VCA. The L_1_-regularized model shrinks coefficients to prevent overfitting of multiple data. Subsequently, atrial fibrillation and lateromedial anulus diameter of the mitral valve exhibited a nonzero coefficient and thus contributed to the significant prediction of EROA underestimation between 2D and 3D echocardiography.

**Supplementary Figure S3. (A**) Comprehensive correlation matrix of important clinical parameters alongside quantitative 2D and 3D colour Doppler echocardiography estimators. Spearman´s ρ is coloured accordingly and significant (p<0.05) coefficients are labelled.

* p<0.05, **p<0.01, ***p<0.001

**Supplementary Figure S4. Two-dimensional colour Doppler echocardiography.** Determination of 2D vena contracta width (VCW) of the mitral regurgitation (MR) jet in biplane 2D colour Doppler dataset. Colour Doppler echocardiography images with 2 orthogonal planes of the mid-systolic jet with Nyquist velocity of 40.3 cm/s. Nyquist limits ranged from n 30 to 50 cm/ and a colour gain was used to adjust colour speckle artifacts.

**Supplementary Figure S5.** **Quantification of 3D EROA.** Assessment of three-dimensional effective regurgitation orifice area (EROA) using multiplanar 3D reconstruction of full-volume colour Doppler images. The 3D EROA was obtained by cropping and calibrating three image planes (x, y, z) through the single-heartbeat data set. 2-Chamber view of the narrowest vena contracta width (VCW) of the mitral regurgitation (MR) jet (x plane, upper left). Image with orthogonal VCW assessment of the MR jet (y plane, upper right). Short-axis view with a perpendicular plane (z plane, lower left) along the jet quantifying the minimal cross-section of the MR regurgitation jet using translation and tilting. Realtime 3D colour full-volume acquisition of 3D EROA (lower right).

**Supplementary Figure S6. Underestimation bias of 2D EROA in functional MR.** Representative images of a patient with severe, functional mitral regurgitation (MR). Two-dimensional image planes of the mid-systolic jet of MR. 2-chamber view (left) and longitudinal view (right) highlighting critical discrepancy between MR jet size leading to underestimation of effective regurgitation orifice area (EROA) due to jet abnormalities in functional MR. In this study we present significant underestimation of EROA by two-dimensional colour Doppler echocardiography when compared to the 3D imaging. The 2D method often underestimates or overestimates the EROA in case of opposed geometric assumptions and mostly functional MR. We found that mitral valve anulus diameter dilation was an independent predictive value for the underestimation of 2D EROA.
